# Anesthesia and Surgery Impair Blood–Brain Barrier and Cognitive Function in Mice

**DOI:** 10.3389/fimmu.2017.00902

**Published:** 2017-08-09

**Authors:** Siming Yang, Changping Gu, Emiri T. Mandeville, Yuanlin Dong, Elga Esposito, Yiying Zhang, Guang Yang, Yuan Shen, Xiaobing Fu, Eng H. Lo, Zhongcong Xie

**Affiliations:** ^1^Key Laboratory of Wound Repair and Regeneration of PLA, College of Life Sciences, General Hospital of PLA, Medical College of PLA, Beijing, China; ^2^Geriatric Anesthesia Research Unit, Department of Anesthesia, Critical Care and Pain Medicine, Massachusetts General Hospital, Harvard Medical School, Charlestown, MA, United States; ^3^Department of Anesthesiology, Shandong Provincial Qianfoshan Hospital, Jinan, China; ^4^Neuroprotection Research, Radiology and Neurology, Massachusetts General Hospital, Harvard Medical School, Charlestown, MA, United States; ^5^Department of Anesthesiology, Perioperative Care and Pain Medicine, New York University School of Medicine, New York, NY, United States; ^6^Department of Psychiatry, Tenth People’s Hospital of Tongji University, Shanghai, China

**Keywords:** anesthesia/surgery, interleukin-6, blood–brain barrier, age, cognition

## Abstract

Blood–brain barrier (BBB) dysfunction, e.g., increase in BBB permeability, has been reported to contribute to cognitive impairment. However, the effects of anesthesia and surgery on BBB permeability, the underlying mechanisms, and associated cognitive function remain largely to be determined. Here, we assessed the effects of surgery (laparotomy) under 1.4% isoflurane anesthesia (anesthesia/surgery) for 2 h on BBB permeability, levels of junction proteins and cognitive function in both 9- and 18-month-old wild-type mice and 9-month-old interleukin (IL)-6 knockout mice. BBB permeability was determined by dextran tracer (immunohistochemistry imaging and spectrophotometric quantification), and protein levels were measured by Western blot and cognitive function was assessed by using both Morris water maze and Barnes maze. We found that the anesthesia/surgery increased mouse BBB permeability to 10-kDa dextran, but not to 70-kDa dextran, in an IL-6-dependent and age-associated manner. In addition, the anesthesia/surgery induced an age-associated increase in blood IL-6 level. Cognitive impairment was detected in 18-month-old, but not 9-month-old, mice after the anesthesia/surgery. Finally, the anesthesia/surgery decreased the levels of β-catenin and tight junction protein claudin, occludin and ZO-1, but not adherent junction protein VE-cadherin, E-cadherin, and p120-catenin. These data demonstrate that we have established a system to study the effects of perioperative factors, including anesthesia and surgery, on BBB and cognitive function. The results suggest that the anesthesia/surgery might induce an age-associated BBB dysfunction and cognitive impairment in mice. These findings would promote mechanistic studies of postoperative cognitive impairment, including postoperative delirium.

## Introduction

Anesthesia and surgery have been shown to induce postoperative cognitive impairment in rodents [(1–10), reviewed in Ref. (11)]. However, the underlying mechanism by which the anesthesia/surgery causes cognitive impairment remains largely to be determined.

The blood–brain barrier (BBB) is composed of blood vessels with endothelial cells with extremely low rates of paracellular vesicular transport (12) and transcellular vesicular transport (transcytosis) [(13–15), reviewed in Ref. (16)]. The BBB provides a safe environment for the central nervous system (CNS), which is free of pathogens and toxins in normal conditions [reviewed in Ref. (17)].

Compromised BBB function might contribute to the cognitive impairment, brain damage, and neurodegenerative disorders, e.g., Alzheimer’s disease [(18, 19), reviewed in Ref. (16, 20, 21)]. Specifically, peripheral inflammation can profoundly affect the function of the CNS, including cognitive impairment and delirium, through a compromised BBB (22–24). Stranahan et al. reported that BBB dysfunction (e.g., increase in BBB permeability) might induce cognitive impairment in obese and diabetic patients by allowing macrophage infiltration and trafficking of interleukin (IL)-1β from peripheral monocytes to the brain (25). Zhang et al. suggested that BBB dysfunction caused CNS inflammation and cognitive impairment (26). Based on these findings, we aimed to assess the effects of the anesthesia/surgery on BBB permeability, the potential underlying mechanisms and cognitive function in the current studies.

Our previous studies have shown that surgery under local anesthesia induces an age-dependent cognitive impairment in mice (6, 7), and anesthetic isoflurane (27) and sevoflurane (28, 29) can increase IL-6 level. Therefore, we also sought to determine whether the anesthesia/surgery-induced change in BBB permeability and cognitive function was associated with the advanced age and dependent on IL-6. Finally, we compared the effects of anesthesia/surgery on the levels of β-catenin, tight junction proteins (claudin, occludin, and ZO-1) [(30), reviewed in Ref. (16)], and adherent junction protein (VE-cadherin, E-cadherin, and p120-catenin) [(31), reviewed in Ref. (16)].

The objective of the current studies was to determine the effects of the anesthesia/surgery on BBB permeability, cognitive function and blood IL-6 levels, and brain levels of tight junction and adherent junction proteins in adult and older mice, which could lead to the further mechanistic investigation of postoperative cognitive impairment, including postoperative delirium. Our hypothesis is that the anesthesia/surgery induces an age-associated and IL-6 dependent increase in BBB permeability, leading to cognitive impairment in mice.

## Materials and Methods

### Mice Surgery and Treatment

All experiments were performed in accordance with the National Institutes of Health guidelines and regulations. The animal protocol was approved by the Massachusetts General Hospital (Boston, MA, USA) Standing Committee on the Use of Animals in Research and Teaching. Efforts were made to minimize the number of animals used. Wild-type C57BL/6J female mice (9-month-old, The Jackson Laboratory, Bar Harbor, ME, USA; and 18-month-old, National Institute of Aging, Bethesda, MD, USA), and 9-month-old IL-6 gene knockout female mice (B6.129S2-Il6tm1Kopf/J, The Jackson Laboratory) were used in the studies.

We only used female mice in the current studies because our previous studies showed that the female mice were more vulnerable to the development of cognitive impairment following the same anesthesia/surgery (32). Mice were randomly assigned to the anesthesia/surgery group or control group by weight. The 18-month-old mice are only available through National Institute of Aging, thus cannot be purchased from same company that provides the 9-month-old mice. Mice in the anesthesia/surgery group had a simple laparotomy under isoflurane anesthesia using the methods described in our previous studies (10, 33). Specifically, we anesthetized each of the mice using 1.4% isoflurane in 100% oxygen in a transparent acrylic chamber. Fifteen minutes after the induction, we moved the mouse out of the chamber. Isoflurane anesthesia was maintained *via* a cone device and one 16-G needle was inserted into the cone near the nose of the mouse to monitor the concentration of isoflurane. We made a longitudinal midline incision from the xiphoid to the 0.5 cm proximal pubic symphysis on the skin, abdominal muscles, and peritoneum. We then sutured the incision layer by layer with 5–0 Vicryl thread. We applied EMLA cream (2.5% lidocaine and 2.5% prilocaine) to the incision site at the end of the procedure, and then every 8 h until the euthanasia of the mice, to treat the pain associated with the incision. The procedure for each mouse usually lasted about 10 min and we put the mouse back into the anesthesia chamber for up to 2 h to receive the rest of the anesthesia consisting of 1.4% isoflurane in 100% oxygen. We used this method because surgery could potentiate the anesthesia neurotoxicity and such combination of anesthesia and surgery had been shown to induce cognitive impairment (10, 33). We maintained the rectal temperature of the mice at 37 ± 0.5°C during the anesthesia/surgery by using DC Temperature Control System (FHC, Bowdoinham, ME, USA). We returned the mice back to their home cage with food and water available *ad libitum* after recovering from the anesthesia. The mice in the control group were placed in their home cages with regular room air for 2 h, which was consistent with the condition of non-surgery patients. Our previous studies found that neither the surgery (6, 7) nor anesthesia with 1.4% isoflurane (34) significantly disturbed the blood pressure and blood gas values of the mice. EMLA could treat the pain associated with the surgery in the mice (6, 7). The treatment of IL-6 antibody was performed as described in previous studies with modification (4). Specifically, each of the 18-month-old mice received the 10 µg IL-6 antibody (eBioscence Inc., San Diego, CA, USA, Cat. Number: 16-7061) at 18 h before the anesthesia/surgery *via* tail vein injection under brief anesthesia (1.4% isoflurane for 5 min). The control mice received saline. We used a single injection of IL-6 antibody because a single injection of TNF-α antibody had been shown to mitigate the surgery-induced cognitive impairment in mice in the studies by Terrando et al. (35).

### Brain Tissue Harvest

We harvested the brain tissues of the mice for the dextran imaging studies and spectrophotometer quantification of dextran. We harvested both cortex and hippocampus for the Western blot analysis. Each of the mice was perfused with phosphate-buffered saline (PBS) for the spectrophotometric quantification of dextran. Specifically, mouse received thoracotomy under brief anesthesia (1.4% isoflurane for 5 min), we inserted a needle to left ventricular of the heart and perfused slowly with sufficient amount of PBS (five times with 30 ml PBS each time) until the PBS exiting from right heart became colorless. We then decapitated the head of each of the mice and harvested the brain tissues. We stored the brain tissues in a −80°C freezer for future analysis.

### Dextran Imaging Studies to Detect BBB Permeability

Dextran was used to measure BBB permeability as described in previous studies with modifications (15). Specifically, 6 h after the anesthesia/surgery, each of the mice was briefly anesthetized with 1.4% isoflurane for 5 min for the injection of dextran. 100 µl 10-kDa dextran tetramethylrhodamine lysine fixable (4 mg/ml, Catalog number: D3312, Invitrogen) was injected into the mouse through the tail vein. Ten minutes after the injection, each of the mice was decapitated; brain tissues (e.g., cortex) were harvested and fixed by 4% paraformaldehyde overnight at 4°C. The brain tissues (e.g., cortex) were cryopreserved in 30% sucrose and frozen in TissueTek OCT (Sakura). The immunohistochemistry to detect BBB permeability was performed as described in the previous studies (15) with modifications. Frozen sections (the thickness of each section: 12 µm) from mouse brain hemispheres were cut and used for the immunohistochemistry staining. These sections were postfixed in 4% PFA at room temperature (20–25°C) for 15 min, washed in PBS, and were blocked with 2% albumin from bovine serum, permeabilized with 0.5% Triton X-100, and incubated with isolectin B4 (1:200; Catalog number: I21411, Molecular Probes, San Francisco, CA, USA) for the immunohistochemistry imaging of blood vessels. Dextran itself has fluorescence for the detection of immunohistochemistry imaging. Finally, the sections were analyzed in mounting medium (Catalog number: ab104139; Abcam, Cambridge, MA, USA) under a 40× objective lens of the fluorescence microscope and photos of the sections were taken. These images were analyzed manually with ImageJ (National Institutes of Health, Bethesda, MD, USA). Coronal cortical sections (12 µm) of the same rostrocaudal position were used for the analysis. The same threshold was used and the same acquisition parameters were applied to all images. An investigator who was blind to the experimental design manually measured the level (combined with area and intensity) of the dextran tracer-positive area found outside the vessel using ImageJ. For each mouse, we obtained five slices from front, middle, and posterior section of the brain tissue (e.g., cortex). Two images per slice were taken. Therefore, 30 images (3 section × 5 slices × 2 images) of each mouse were counted. There were three mice in control group and three mice in the anesthesia/surgery group. Thus, the quantification of the images was based on these 90 images per group.

### Spectrophotometric Quantification of Dextran to Detect BBB Permeability

Spectrophotometric quantification of 10-kDa fluoro-ruby-dextran tracer (555/580) (4 mg/ml, Catalog number: D1817, Invitrogen) from cortex extracts was used as described in previous studies (15, 36). The 100 µl dextran tracer was injected into each of the mice *via* the tail vein under brief anesthesia (1.4% isoflurane for 5 min) at 1 h after the anesthesia/surgery. 16 h after the injection of the tracer, each of the mice was cardiac perfused as described in the Section “Brain Tissues Harvest.” Brain tissues (e.g., cortex) were then harvested and the 10-kDa fluoro-ruby-dextran tracer (555/580) from cortex extracts was determined by Spectrophotometric measurement.

### Enzyme-Linked Immunosorbent Assay (ELISA) Determination of IL-6

The mouse IL-6 Immunoassay kit (Catalog number: M6000B, R&D Systems) was used to determine the levels of IL-6 in mouse blood as described by the protocol associated with the immunoassay kit. Briefly, a monoclonal antibody specific for mouse IL-6 was coated onto the microplates. Wells were incubated for 2 h at room temperature with test samples (serum) and washed for five times. Then, 100 µl of mouse IL-6 conjugate was added to each well and incubated for another 2 h and we repeated the washing. Finally, wells were incubated in 100 µl of substrate solution for 30 min and stopped with stop solution (100 µl). Determination of the optical density of each well was set at 450 nm and corrected at 570 nm.

### Western Blot Analysis

Western blot analysis was performed using the methods described in our previous studies (8). Cortex and hippocampus tissues were harvested from 18-month-old mice at 6, 12, and 24 h after the anesthesia/surgery. Anti-β-catenin antibody (92 kDa, Cat: #9562, 1:1,000 dilution, Cell signaling, Danvers, MA, USA), anti-phosphorylated β-catenin antibody (Ser33/37/Thr41, 92 kDa, Cat: #9561, 1:1,000 dilution, Cell signaling), anti-claudin-1 antibody (19 kDa, Cat: ab15098, 1:1,000 dilution, Abcam, Cambridge, MA, USA), anti-occludin antibody (59 kDa, Cat: ab167161, 1:1,000 dilution, Abcam), anti-ZO-1 antibody (250 kDa, Cat: PA5-28858, 1:500 dilution, Thermo Fisher Scientific, Rockford, IL, USA), and anti-VE-cadherin (115 kDa, Cat: ab33168, 1 µg/ml, Abcam), anti-E-cadherin antibody (135 kDa, Cat: #5296, 1:1,000 dilution, Cell signaling) were used to detect the level of the proteins in the cortex and hippocampus of the 18-month-old mice. Samples in different groups were loaded and β-actin was used to normalize (e.g., determining the ratio of β-catenin to β-actin amount) protein levels and control for loading differences in the total protein amount. The quantification of the Western blot was performed as described in previous study (8), we presented changes in protein levels in hippocampus or cortex treated with anesthesia/surgery as a percentage of those in the control group. 100% of protein level changes refer to control levels for the purpose of comparison to experimental conditions. Signal intensity was analyzed using a Bio-Rad (Hercules, CA, USA) image program.

### Morris Water Maze (MWM)

**Figure 1:**
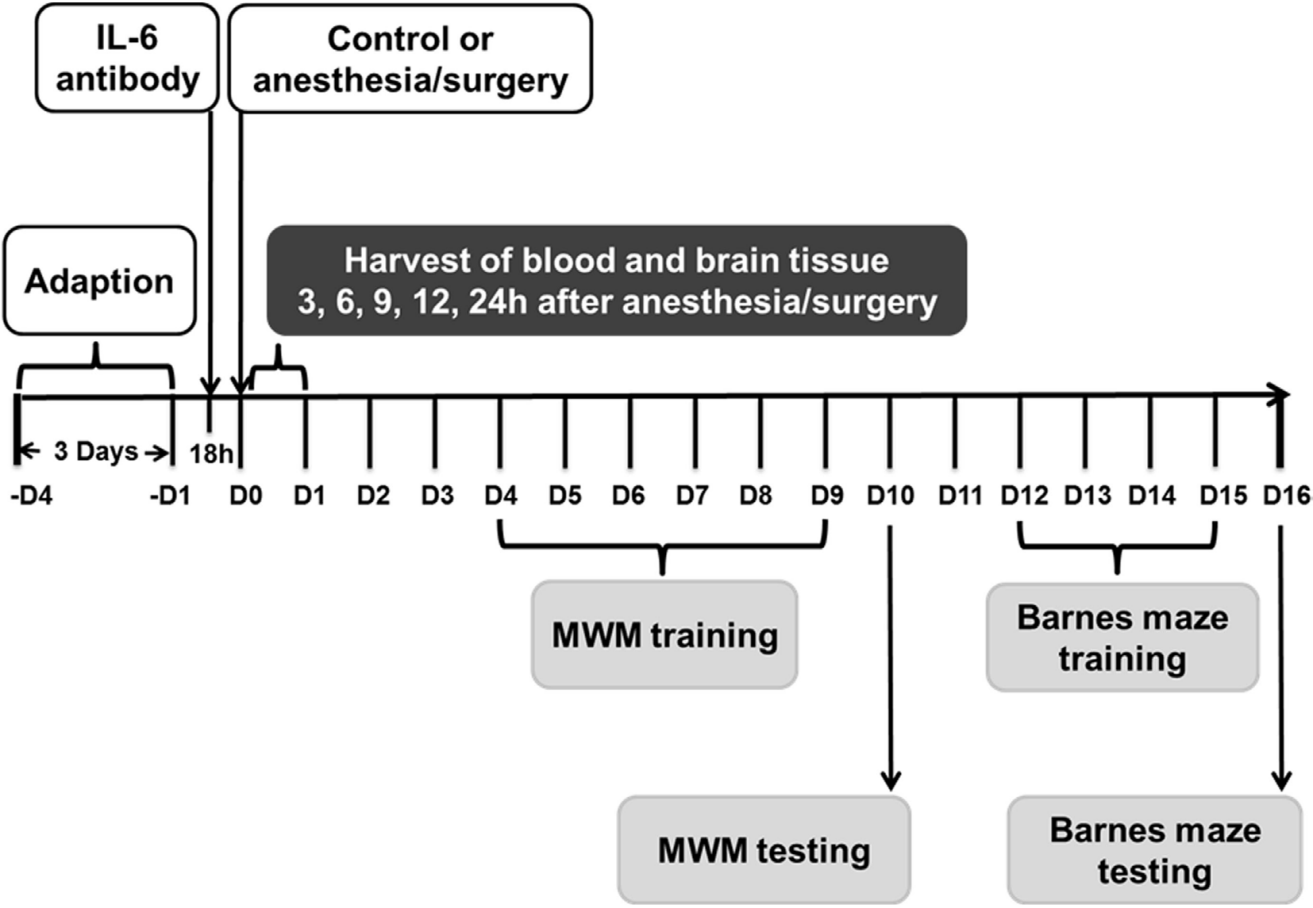
Experimental design. Nine- and eighteen-month-old mice received the anesthesia/surgery or control condition. The blood and brain tissues were harvested at various time points after the anesthesia/surgery. Different group of mice were used for MWM and Barnes maze studies. Interleukin (IL)-6 antibody was used in the intervention studies. MWM, Morris water maze.

Morris water maze was carried out to assess spatial learning and memory function as previously described (7, 28). The blind procedure was not possible in the MWM studies because of the appearance of the abdominal wound in the mice. Briefly, all mice were trained to swim to a hidden platform in four trials per day for 6 days (days 1–6) starting 3 days after the anesthesia/surgery (Figure 1). Each of the mice was given 90 s to find the platform and allowed to stay there for 15 s before being removed from the platform. Mice would be guided to the platform and allowed to stay on the platform for 15 s if they could not find the platform within 90 s. The platform was placed in the target quadrant in trials within one MWM test, but the starting points were random for each mouse. We measured the time it took for each mouse to reach the platform (escape latency) and used this as the learning score. On the seventh day, we repeated the training same as the previous training (days 1–6). Two hours after the training, we removed the platform and assessed the memory of each mouse by measuring the number of times the mouse crossed the platform area with the same cue (memory score). We compared the learning and memory score and swimming speed between the mice in the control group and the anesthesia/surgery group.

### Barnes Maze

Barnes maze test was performed by using the methods described in other studies (37–40) with modifications. The blind procedure was not possible in the Barnes maze studies because of the appearance of the abdominal wound in the mice. Barnes maze with a circular open platform (about 90-cm diameter) was located in a quiet area. It had 20 equally spaced holes (one of these holes connects with a small dark recessed chamber called escape box) and was surrounded by a dark curtain with four simple colored-paper shapes (square, circle, triangle, and star) as markers (Stoelting, Wood Dale, IL, USA). A video camera that could capture the entire platform was right above the platform and connected to the Any-Maze animal tracking system software (Stoelting Co., Wood Dale, IL, USA) as described in previous studies (38, 41, 42). The movement parameters, including escape latency (the total time to find the escape box), escape distance (the total path length of distance traveled), escape speed (mean speed), escape errors (the total error holes searched), and time in target quadrant [the percentage of time spent in the target quadrant, time in target quadrant (%)], of the mouse before finding the escape box in both training and test were monitored and analyzed *via* the video camera. The Barnes maze test in the current studies included Barnes maze training test (days 12–15 after the anesthesia/surgery) and Barnes maze test (16 days after the anesthesia/surgery) (Figure 1). On day 11 after the anesthesia/surgery, all of the mice were habituated to the maze. The mouse was placed in the escape box for 2 min and then placed directly in the hole that led to the escape box for another 4 min. Finally, the mouse was placed under a bucket in the center of the circular platform and motivated to escape under the bright light (200 W) and noise (85 dB) stimulation. Mouse was gently guided to the hole connecting to the escape box when it did not go into the escape box 3 min after the light and noise stimulation. Immediately after the mouse entered the tunnel between the hole and the escape box, the buzzer was turned off. Each mouse was allowed to remain in the escape box for 1 min and then removed and placed back to the home cage. The Barnes maze training test (days 12–15 after the anesthesia/surgery) consisted of two trials (3 min each trial and 15 min between the trials) for 4 days. In each trial, the mouse was placed under a bucket in the center of the circular platform for 10 s and was allowed to escape under the same stimulation of light and aversive noise. Once reaching the escape box, the mouse was allowed to remain in the escape box for 1 min. The mouse was then removed and placed back to the home cage for 15 min of rest period before returning back for another trial. Between each test, the Barnes maze was cleaned with 75% alcohol solution to avoid olfactory cues. After the training period, the mice had the Barnes maze test on day 16 after the anesthesia/surgery (Figure 1). The increases in escape latency, escape distance, escape errors, and decrease in time in target quadrant in the Barnes maze suggests cognitive impairment of the mice (37–40). The decreases in escape speed suggest impairment of locomotor activity. We used the percentage of time in the target quadrant rather than the target preference in the current studies according to the previous studies (41, 43).

### Statistics

Data of escape latency, escape distance, escape speed, escape errors, and time in target quadrant were expressed as means ± SEM. Data of other variables were expressed as means ± SD. The number of samples was 3 per group for ELISA studies, 4–5 per group for the spectrophotometer quantification of dextran, 90 per group for the immunohistochemistry imaging study, 10 per group for behavior test, and 6 per group for the Western blot studies. In the Barnes maze and Water maze studies, interaction between time and group factors in a two-way ANOVA with repeated measurements was used to analyze the difference of memory curves (e.g., based on escape latency). *Post hoc* analyses (Bonferroni) were used to compare the difference in all behavior test parameters for each testing day. In the biochemistry studies, one-way ANOVA or two-way ANOVA was used to determine the interaction between group and treatment on the levels of β-catenin, claudin, occludin, ZO-1, VE-cadherin, E-cadherin, and p120-catenin followed by Bonferroni test for the comparisons. All the protein levels were presented as a percentage of those of the control group. *P*-values less than 0.05 were considered statistically significant. Prism 6 software (Graph Pad Software, Inc., La Jolla, CA, USA) was used to analyze the data.

## Results

### Anesthesia/Surgery Increases Extravascular Dextran Level in Mouse Brain in Age-Associated Manner

**Figure 2:**
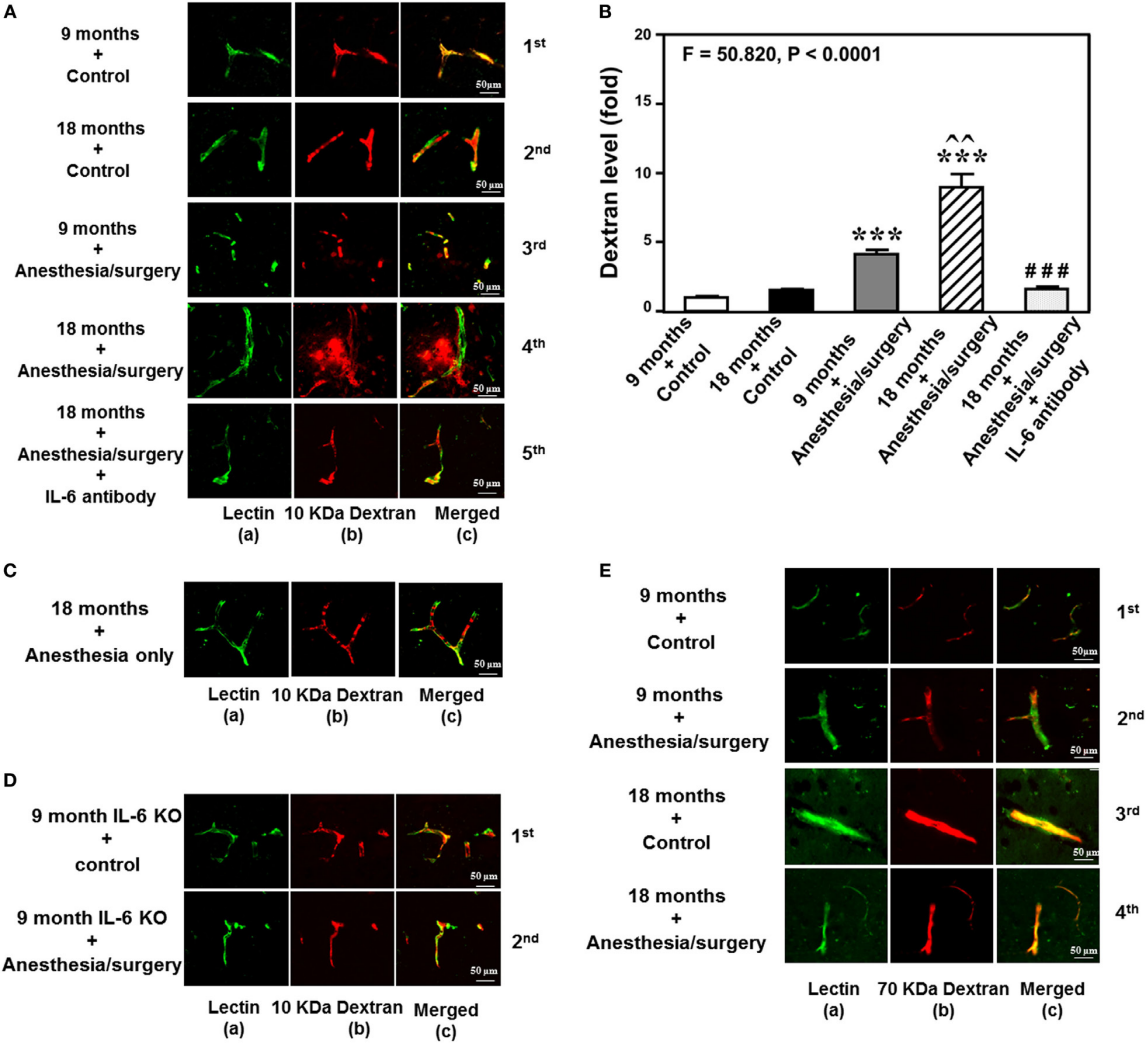
Anesthesia/surgery increases blood–brain barrier permeability of dextran in mice in an age-associated manner. **(A)** Immunostaining of blood vessels (lectin, green, column *a*) and dextran (10-kDa dextran, red, column *b*) of the brain section following the control condition in 9-month-old mice (the first row), the control condition in 18-month-old mice (the second row), the anesthesia/surgery in 9-month-old mice (the third row), the anesthesia/surgery in 18-month-old mice (the fourth row), and the anesthesia/surgery in 18-month-old mice pre-treated with IL-6 antibody (the fifth row). Column *c* is the merged image of columns *a* and *b*. The red spots (non-overlap area) in column *c* indicate the dextran that is not inside the blood vessel (extravascular dextran). *N* = total of 90 slides from 3 mice in each group. **(B)** Quantification of the immunostaining images shows that the anesthesia/surgery in 9-month-old mice (gray bar) increases the extravascular dextran level in the mouse brain tissues as compared to that in the control condition in 9-month-old mice (white bar). The anesthesia/surgery in 18-month-old mice (net bar) increases extravascular dextran level in the mouse brain as compared to that in the control condition in 18-month-old mice (black bar) and that in anesthesia/surgery condition in 9-month-old-mice (gray bar). Treatment with IL-6 antibody (dot bar) attenuates the anesthesia/surgery-induced increase in the extravascular dextran level in the brain tissues of 18-month-old mice (net bar). **(C)** Immunostaining of blood vessels (lectin, green, column *a*) and dextran (10-kDa dextran, red, column *b*) of the brain section following anesthesia only in one 18-month-old mouse. Column *c* is the merged image of columns *a* and *b*. Anesthesia only does not increase the extravascular dextran level in the brain tissues of the 18-month-old mouse. The image represents one mouse. The studies were repeated twice in another two mice. **(D)** Immunostaining of blood vessels (lectin, green, column *a*) and dextran (10-kDa dextran, red, column *b*) of the brain section following anesthesia/surgery in one 9-month-old IL-6 gene knockout mouse. Column *c* is the merged image of columns *a* and *b*. The red spots (non-overlap area) in column *c* indicate the dextran that is not inside the blood vessel (extravascular dextran). Anesthesia/surgery does not significantly increase the extravascular dextran level in the brain tissues of the 9-month-old IL-6 gene knockout mouse. The study was repeated in another mouse. **(E)** Immunostaining of blood vessels (lectin, green color, column *a*) and 70-kDa dextran (red, column *b*) of the brain section following the control condition in 9-month-old mice (the first row), the anesthesia/surgery in 9-month-old mice (the second row), the control condition in 18-month-old mice (the third row), and the anesthesia/surgery in 18-month-old mice (the fourth row). Column *c* is the merged image of columns *a* and *b*. The red spots (non-overlap area) in column *c* indicate the 70-kDa dextran that is not inside the blood vessel (extravascular dextran). The anesthesia/surgery does not significantly increase the extravascular 70-kDa dextran level in the brain tissues of the 9-month-old mouse and 18-month-old mouse as compared to control condition. The image represents one mouse. The studies were repeated in another two mice. IL, interleukin.

The details of the experimental design and performance were summarized in Figure 1. We employed immunohistochemistry staining of blood vessels (green color) and 10-kDa dextran (red color) to determine whether the abdominal surgery under isoflurane anesthesia (anesthesia/surgery) could increase the BBB permeability in mice. As compared to control condition (Figure 2A, first row), the anesthesia/surgery (Figure 2A, third row) increased the extravascular 10-kDa dextran level in the brain tissues of the 9-month-old mice. The quantification of the immunohistochemistry image showed that the anesthesia/surgery (Figure 2B, gray bar) increased the extravascular 10-kDa dextran level in the brain tissues of mice as compared to the control condition (Figure 2B, white bar) (*P* < 0.001, one-way ANOVA and Bonferroni test). These data suggest that the anesthesia/surgery was able to increase BBB permeability of 10-kDa dextran in the 9-month-old mice.

We then assessed the effects of the anesthesia/surgery on extravascular 10-kDa dextran level in the brain tissues of the 18-month-old mice. We found that the anesthesia/surgery induced a greater increase in the extravascular 10-kDa dextran level in the brain tissues of the 18-month-old mice (Figure 2A, fourth row) as compared to the control condition (Figure 2A second row) or the anesthesia/surgery in the 9-month-old mice (Figure 2A, third row). The quantification of the immunohistochemistry images showed that the anesthesia/surgery in 18-month-old mice (Figure 2B, net bar) increased the extravascular 10-kDa dextran level in the brain tissues of mice as compared to the control condition (Figure 2B, black bar) (*P* < 0.001, one-way ANOVA and Bonferroni test) or the anesthesia/surgery condition in the 9-month-old mice (Figure 2B, gray bar) (*P* < 0.01, one-way ANOVA and Bonferroni test). These data suggest that the anesthesia/surgery might induce a greater BBB permeability of 10-kDa dextran in the 18-month-old mice.

Finally, we found that the treatment with IL-6 antibody in the 18-month-old mice (Figure 2A, fifth row, and Figure 2B, dot bar) was able to attenuate the anesthesia/surgery-induced increase in BBB permeability of 10-kDa dextran (Figure 2A, fourth row, and Figure 2B, net bar). Anesthesia only in the 18-month-old mice did not significantly increase the BBB permeability of 10-kDa dextran (Figure 2C) as compared to the control condition in the 18-month-old mice (Figure 2A, second row). This image represents one mouse, but the study was repeated in another two mice. Moreover, we employed an IL-6 KO mouse to further assess the effects of IL-6 on the anesthesia/surgery-induced increase in BBB permeability. We found that there was no significant difference in extravascular 10-kDa dextran level in the brain tissues of the 9-month-old IL-6 knockout mouse between control condition and anesthesia/surgery condition (Figure 2D). The image represents one mouse, but the studies were repeated in another mouse. Taken together, these data suggest that the anesthesia/surgery-induced increase in BBB permeability to 10-kDa dextran would be dependent on IL-6 level.

Notably, the same anesthesia/surgery did not significantly increase BBB permeability to 70-kDa dextran in 9- or 18-month-old mice (Figure 2E). The image represents one mouse, but the study was repeated in another two mice. These data suggest that the anesthesia/surgery may only increase BBB permeability to 10-kDa, but not 70-kDa, dextran.

Finally, the findings that the anesthesia/surgery did not increase the BBB permeability to 10-kDa dextran in IL-6 KO mice and in the wild-type mice pre-treated with IL-6 antibody suggest that the perfusion itself would not increase the BBB permeability to 10-kDa dextran.

### Anesthesia/Surgery Increases Extravascular Level of Dextran in Mouse Brain

**Figure 3:**
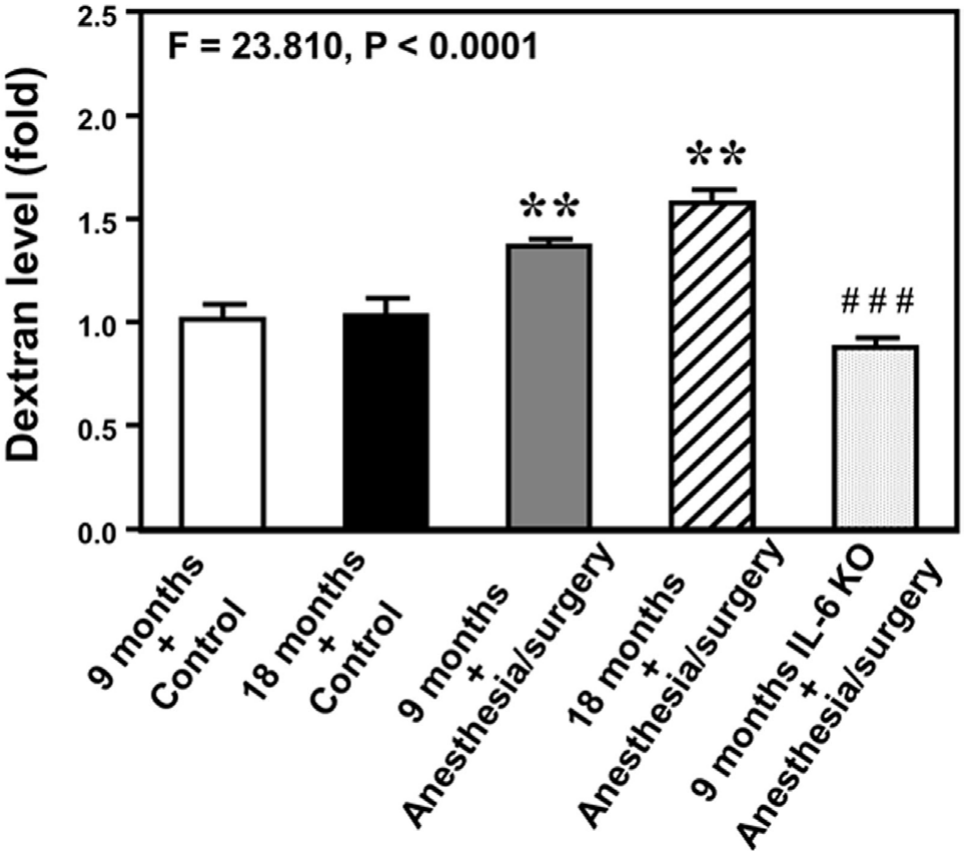
Anesthesia/surgery increases blood–brain barrier permeability of dextran in mice. Spectrophotometric quantification of brain dextran (10 kDa) level. The anesthesia/surgery in 9-month-old mice (gray bar) increases brain dextran level as compared to that in the control condition of 9-month-old mice (white bar). Anesthesia/surgery in 18-month-old mice (net bar) increases brain dextran level as compared to that in the control condition of 18-month-old mice (black bar). Knockout of interleukin (IL)-6 gene (dot bar versus gray bar) attenuates the anesthesia/surgery-induced increase in dextran levels. *N* = 4 in the control condition group and *N* = 5 in the anesthesia/surgery group.

Next, we performed spectrophotometer quantification to further assess whether anesthesia/surgery could increase the extravascular level of 10-kDa dextran in brain tissues of mice. We found that the anesthesia/surgery increased the extravascular level of 10-kDa dextran in the brain tissues of 9-month-old mice (Figure 3, gray bar) as compared to the control condition in the 9-month-old mice (Figure 3, white bar) (*P* < 0.01, one-way ANOVA and Bonferroni test). Similarly, the anesthesia/surgery increased the extravascular level of 10-kDa dextran in the brain tissues of 18-month-old mice (Figure 3, net bar) as compared to the control condition in the 18-month-old mice (Figure 3, black bar) (*P* < 0.01, one-way ANOVA and Bonferroni test). However, anesthesia/surgery did not increase the extravascular level of 10-kDa dextran in the brain tissues of 9-month-old IL-6 KO mice (Figure 3 dot bar versus gray bar versus white bar) (*P* < 0.001, one-way ANOVA and Bonferroni test). These data further suggest that the anesthesia/surgery is able to increase BBB permeability and the effects could be dependent on IL-6 level.

### Anesthesia/Surgery Increases Blood IL-6 Level in Mice

**Figure 4:**
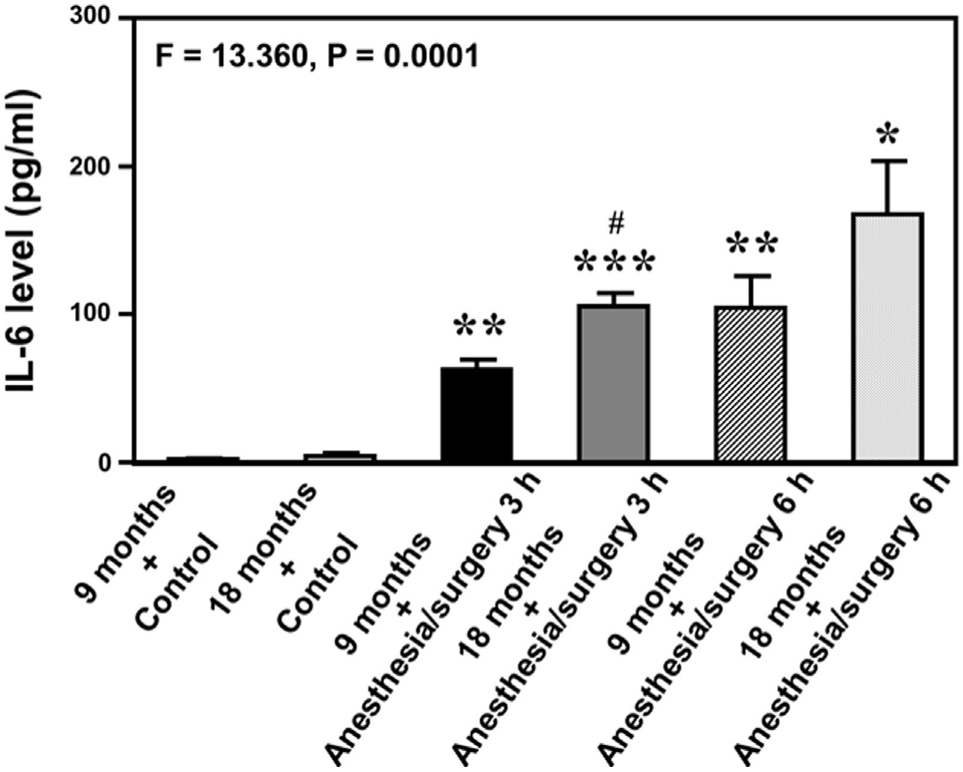
Anesthesia/surgery increases blood IL-6 level in 9- and 18-month-old mice. The anesthesia/surgery increases blood IL-6 level in 9-month-old (black bar and net bar) and 18-month-old (gray bar and dot bar) mice at 3 (middle two bars) and 6 (right two bars) hours after the anesthesia/surgery as compared to the control condition (left two bars). The anesthesia and surgery induces a significant increase in blood IL-6 levels in the 18-month-old mice as compared to that in the 9-month-old mice at 3 and 6 h after the anesthesia/surgery. *N* = 3 in the control condition group and *N* = 3 in the anesthesia/surgery group. IL, interleukin.

Given the findings that the anesthesia/surgery might induce an IL-6-dependent increase in BBB permeability, next we asked whether the anesthesia/surgery could increase blood IL-6 level in the mice. We found that the anesthesia/surgery significantly increased blood IL-6 level as compared to control condition (*left two bars*) at 3 (*middle two bars*) and 6 (*right two bars*) hours after the anesthesia/surgery in both 9-month-old (*black* and *net bar*) and 18-month-old (*gray* and *dot bar*) mice (*F* = 13.36, *P* = 0.0001, one-way ANOVA, Figure 4). Note that the anesthesia/surgery-induced a larger increase in blood IL-6 levels in the 18-month-old mice as compared to that in the 9-month-old mice at 3 h after the anesthesia/surgery (*gray bar* versus *black bar, P* < 0.05). The anesthesia/surgery also induced a greater increase in blood IL-6 levels in the 18-month-old mice as compared to that in the 9-month-old mice at 6 h after the anesthesia/surgery (*net bar* versus *dot bar*). However, the difference did not reach to a statistically significant level. These data suggest that the anesthesia/surgery could induce an age-associated increase in blood IL-6 level in the mice, which would contribute to the anesthesia/surgery-induced increase in BBB permeability in the brain tissues of the mice.

### Anesthesia/Surgery Induces Cognitive Impairment in an Age-Associated Manner

**Figure 5:**
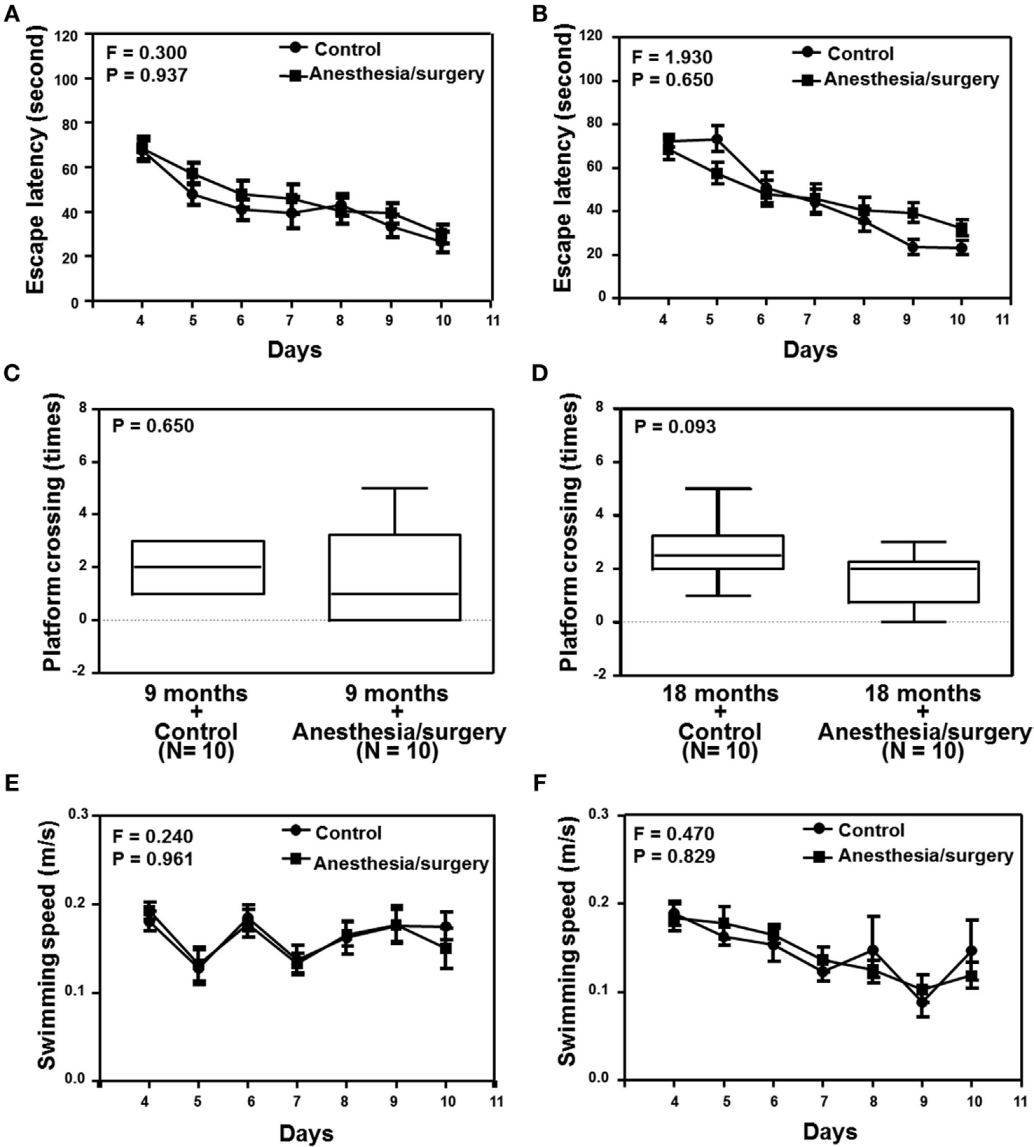
Effects of anesthesia/surgery on cognitive function in 9- and 18-month-old mice tested in MWM. The anesthesia/surgery does not significantly change the escape latency **(A)**, platform crossing times **(C)**, and swimming speed **(E)** of MWM as compared to the control condition in the 9-month-old mice. The anesthesia/surgery does not significantly change the escape latency **(B)** and swimming speed **(F)** of MWM as compared to the control condition in the 18-month-old mice. However, the anesthesia/surgery induces a borderline increase (*P* = 0.093) in platform crossing times **(D)** of MWM as compared to the control condition in the 18-month-old mice. *N* = 10 in the control condition group and *N* = 10 in the anesthesia/surgery group. MWM, Morris water maze.

Given the findings that the anesthesia/surgery increased BBB permeability in an age-associated manner, next we investigated the functional relevance of these findings. Two-way ANOVA with repeated measurement showed no interaction of treatment (control condition and anesthesia/surgery) and time (days 1–7) on escape latency in the 9-month-old mice (Figure 5A, *F* = 0.300, *P* = 0.937). The anesthesia/surgery did not significantly change the platform crossing times (Figure 5C, *P* = 0.464, Mann–Whitney test) or swimming speed (Figure 5E, *F* = 0.240, *P* = 0.961, two-way ANOVA with repeated measurement) as compared to the control condition in the 9-month-old mice. Two-way ANOVA with repeated measurement showed no interaction of treatment (control condition and anesthesia/surgery) and time (days 1–7) on escape latency in the 18-month-old mice (Figure 5B, *F* = 1.930, *P* = 0.650). The anesthesia/surgery induced a borderline increase in the platform crossing times (Figure 5D, *P* = 0.093, Mann–Whitney test) as compared to the control condition in the 18-month-old mice. The anesthesia/surgery did not significantly change the swimming speed (Figure 5F, *F* = 0.240, *P* = 0.961, two-way ANOVA with repeated measurement) as compared to the control condition in the 18-month-old mice.

**Figure 6:**
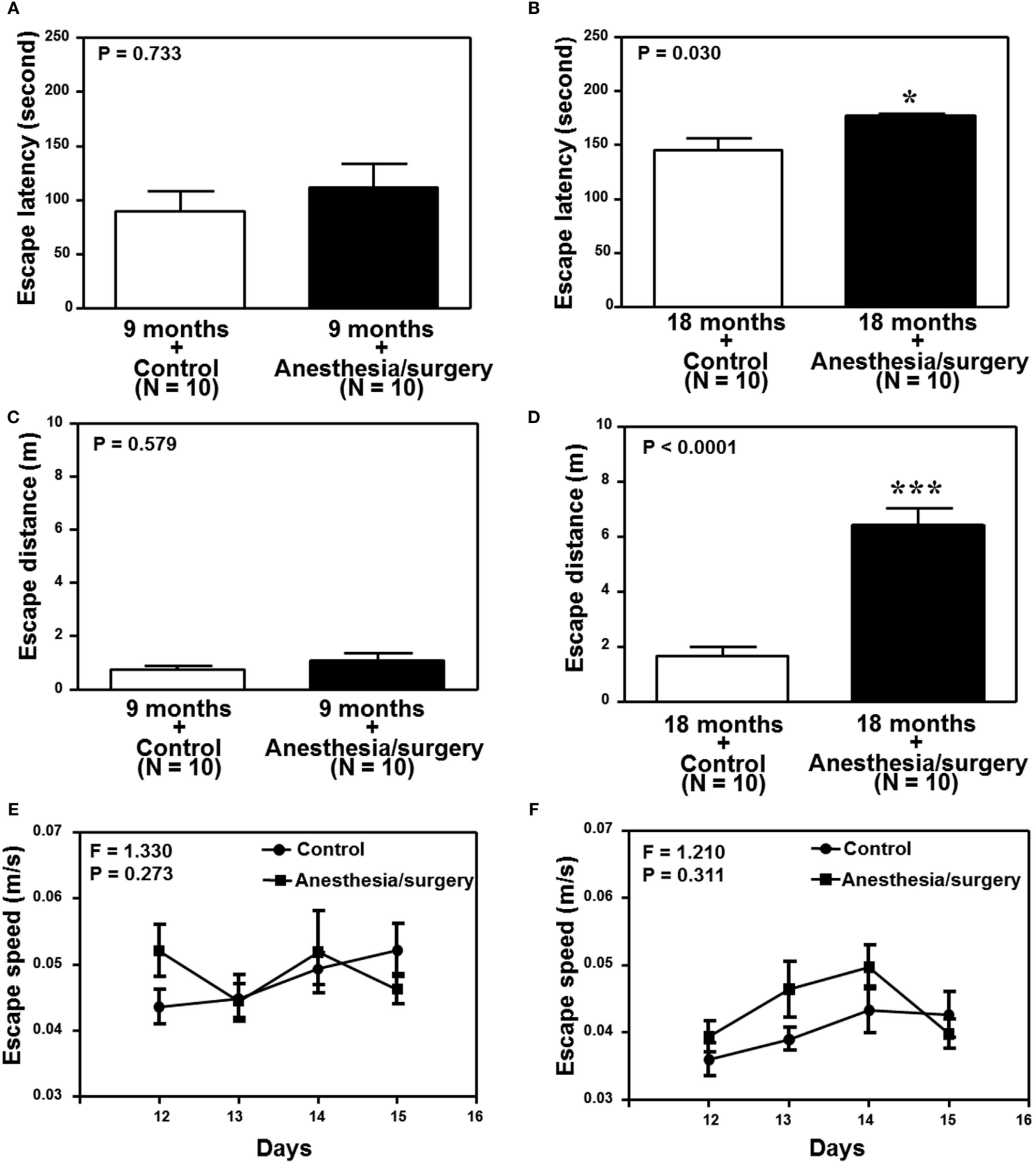

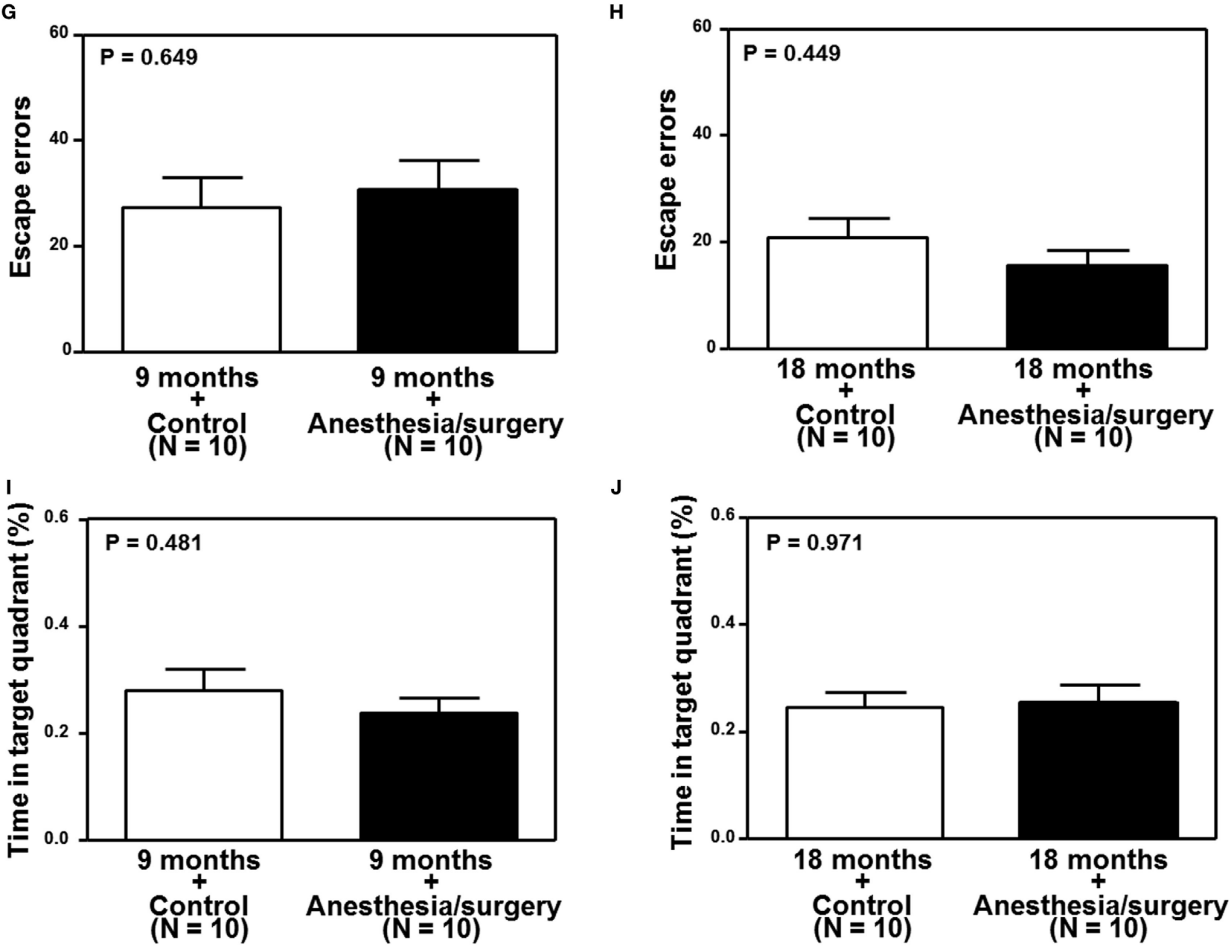
Effects of anesthesia/surgery on cognitive function in 9- and 18-month-old mice tested in Barnes maze. The anesthesia/surgery does not significantly change the escape latency **(A)**, escape distance **(C)**, escape speed **(E)**, escape errors **(G)**, and time in target quadrant **(I)** of Barnes maze as compared to the control condition in the 9-month-old mice. However, in the 18-month-old mice, the anesthesia/surgery significantly increases escape latency **(B)** and escape distance **(D)** of Barnes maze as compared to the control condition. The anesthesia/surgery does not significantly change the escape speed **(F)**, escape errors **(H)**, and time in target quadrant **(J)** of Barnes maze as compared to the control condition in the 18-month-old mice. *N* = 10 in the control condition group and *N* = 10 in the anesthesia/surgery group.

We found that the anesthesia/surgery did not significantly change the escape latency (Figure 6A), escape distance (Figure 6C), escape speed (Figure 6E), escape errors (Figure 6G), and time in target quadrant (Figure 6I) as compared to the control condition in the 9-month-old mice. However, the anesthesia/surgery significantly increased the escape latency (Figure 6B, *P* = 0.0296, Student’s *t*-test) and escape distance (Figure 6D, *P* < 0.0001, Student’s *t*-test) as compared to the control condition in the 18-month-old mice. The anesthesia/surgery did not significantly change the escape speed (Figure 6F), escape errors (Figure 6H), and time in target quadrant (Figure 6J) as compared to the control condition in the 18-month-old mice. Taken together, these data suggest that the anesthesia/surgery could induce an age-associated cognitive impairment in mice.

### Anesthesia/Surgery Decreases Levels of Cell Junction Proteins

**Figure 7:**
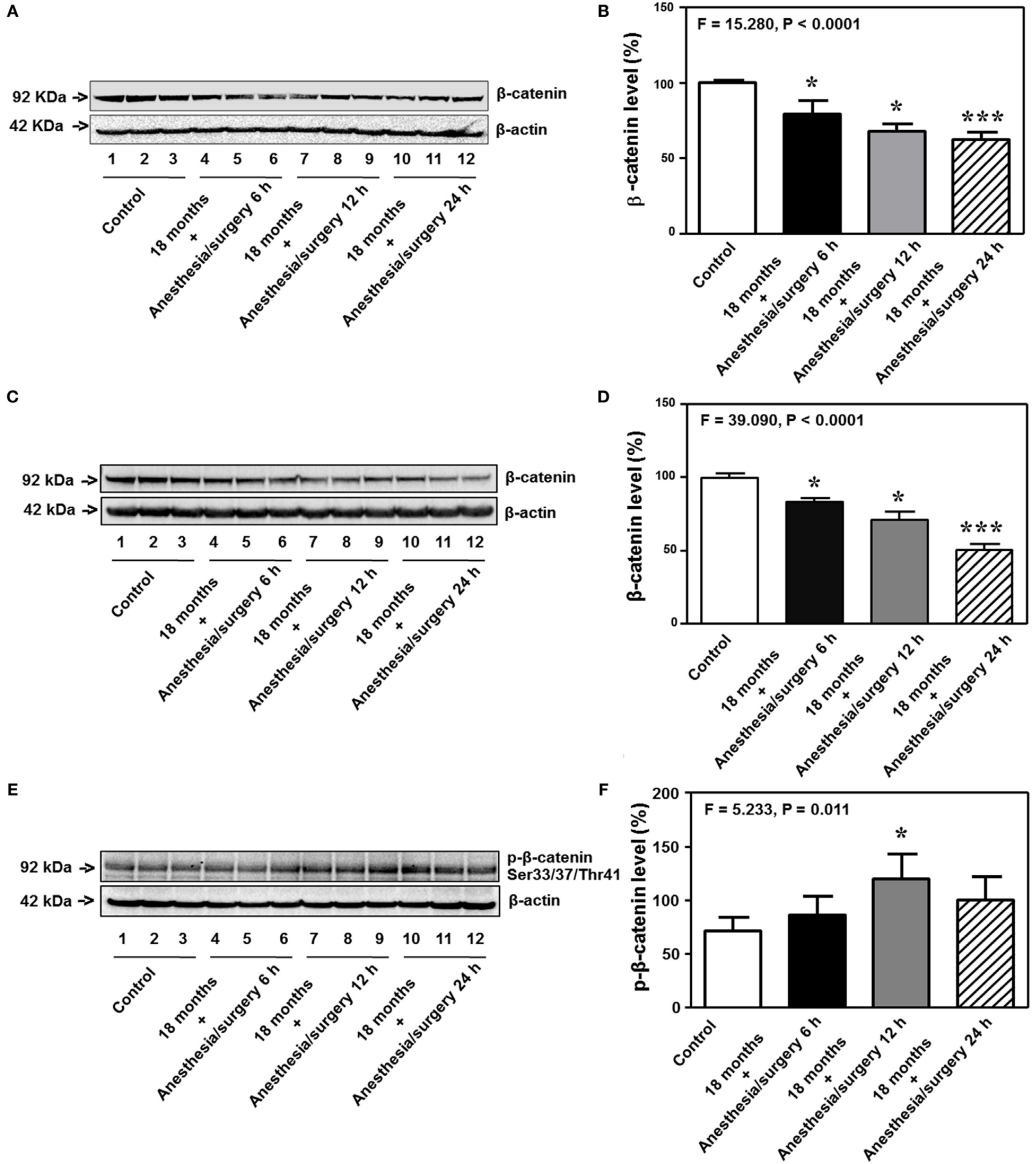

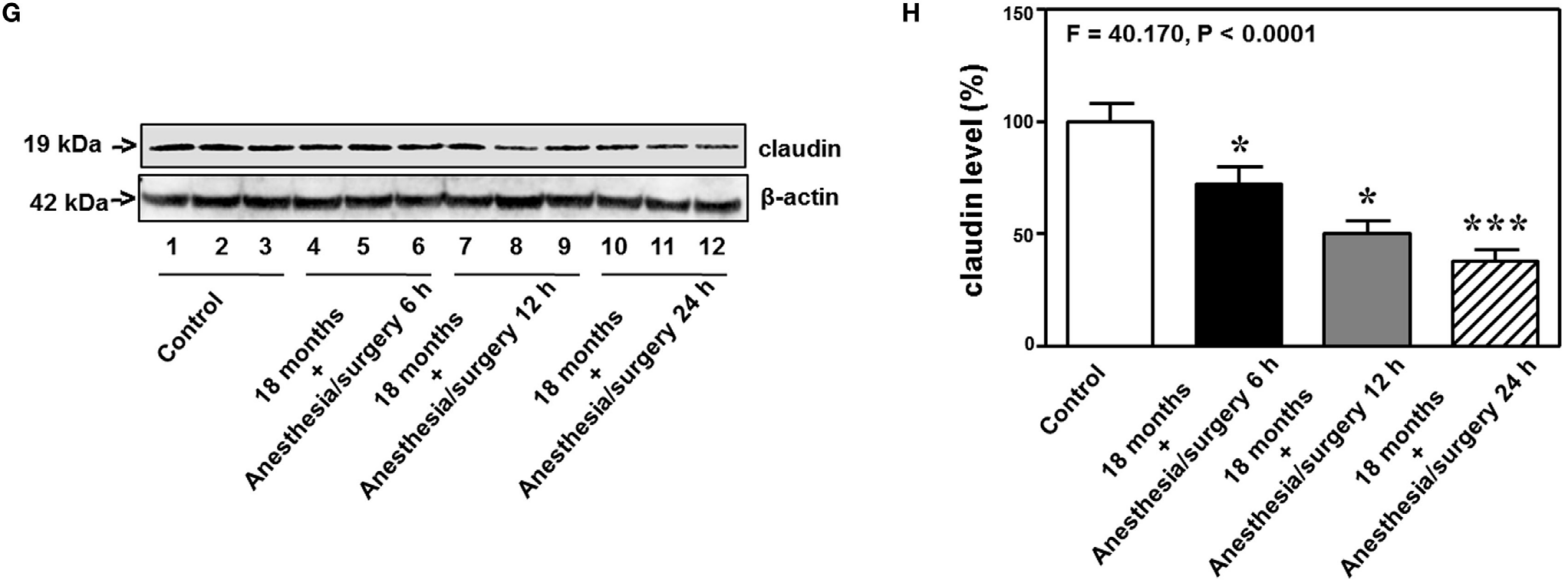
Anesthesia/surgery decreases levels of cell junction proteins. The anesthesia/surgery decreases β-catenin levels in the cortex of 18-month-old mice at 6, 12, and 24 h after the anesthesia/surgery as compared to the control condition **(A,B)**. The anesthesia/surgery decreases β-catenin levels in the hippocampus of 18-month-old mice at 6, 12, and 24 h after the anesthesia/surgery as compared to the control condition **(C,D)**. The anesthesia/surgery increases the phosphorylated β-catenin levels in the hippocampus of 18-month-old mice at 6, 12, and 24 h after the anesthesia/surgery as compared to the control condition **(E,F)**. The anesthesia/surgery reduces the level of claudin in the hippocampus of 18-month-old mice at 6, 12, and 24 h compared to control condition **(G,H)**. *N* = 6 in control or anesthesia/surgery group.

Previous studies in rodents have shown that BBB permeability is associated with the alteration of cell junction protein in cerebral endothelial cells [reviewed in Ref. (16)]. Given the findings that the anesthesia/surgery was able to increase BBB permeability to small, but not big, molecules, next, we compared the effects of the anesthesia/surgery on the levels of β-catenin, tight junction proteins claudin, occludin, and ZO-1, and adherent junction proteins VE-cadherin, E-cadherin, and p120-catenin in cortex and hippocampus of 18-month-old mice. Quantitative Western blot showed that the anesthesia/surgery significantly decreased the level of β-catenin in cortex (*F* = 15.280, *P* < 0.0001, one-way ANOVA, Figures 7A,B) and hippocampus (*F* = 39.090, *P* < 0.0001, one-way ANOVA, Figures 7C,D) as compared to control condition at 6, 12, and 24 h after the anesthesia/surgery in 18-month-old mice. Moreover, quantitative Western blot showed that the anesthesia/surgery increased the phosphorylated β-catenin levels at 6, 12, and 24 h after the anesthesia/surgery as compared to control condition (*F* = 5.233, *P* = 0.011, one-way ANOVA, Figures 7E,F). Note that the antibody (92 kDa, Cat: #9561, 1:1,000 dilution, Cell signaling, Danvers, MA, USA) we used specifically detected the phosphorylated β-catenin (Ser33/37/Thr41) levels in the cytosol (44, 45). Finally, the anesthesia/surgery decreased levels of claudin as compared to control condition at 6, 12, and 24 h after the anesthesia/surgery in hippocampus of 18-month-old mice (*F* = 40.170, *P* < 0.0001, one-way ANOVA, Figures 7G,H).

Interestingly, the anesthesia/surgery did not significantly change the levels of the adherent junction proteins (VE-cadherin, E-cadherin, and p120-catenin) in cortex or hippocampus of 18-month-old mice as compared to the control condition at 6, 12, and 24 h after the anesthesia/surgery (Figure S1 in Supplementary Material). These data suggest that the anesthesia/surgery may specifically regulate the levels of tight junction proteins but not adherent junction proteins in present experiments.

### IL-6 Antibody and KO of IL-6 Attenuates the Anesthesia/Surgery-Induced Reduction in the Levels of Cell Junction Proteins

**Figure 8:**
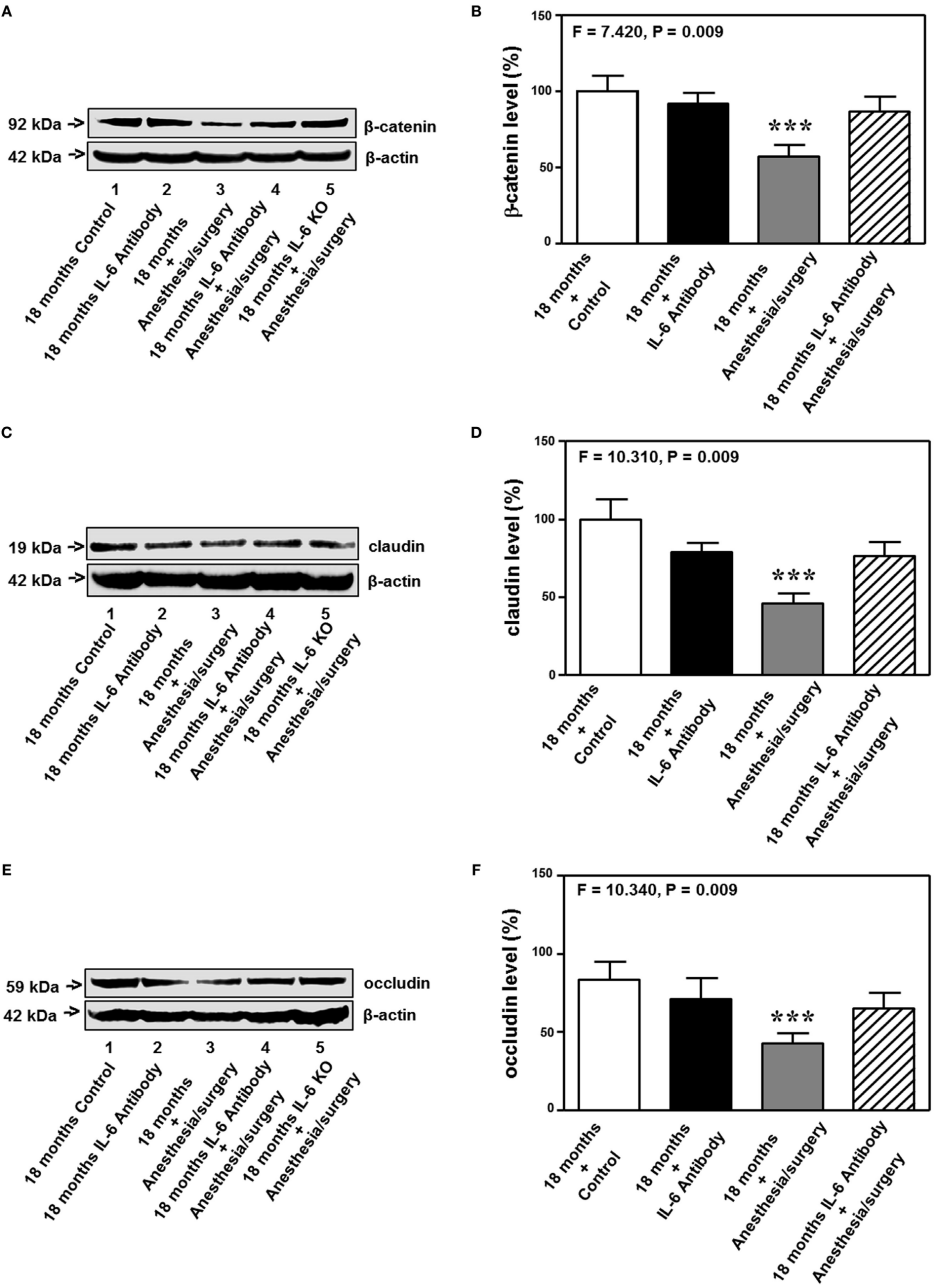

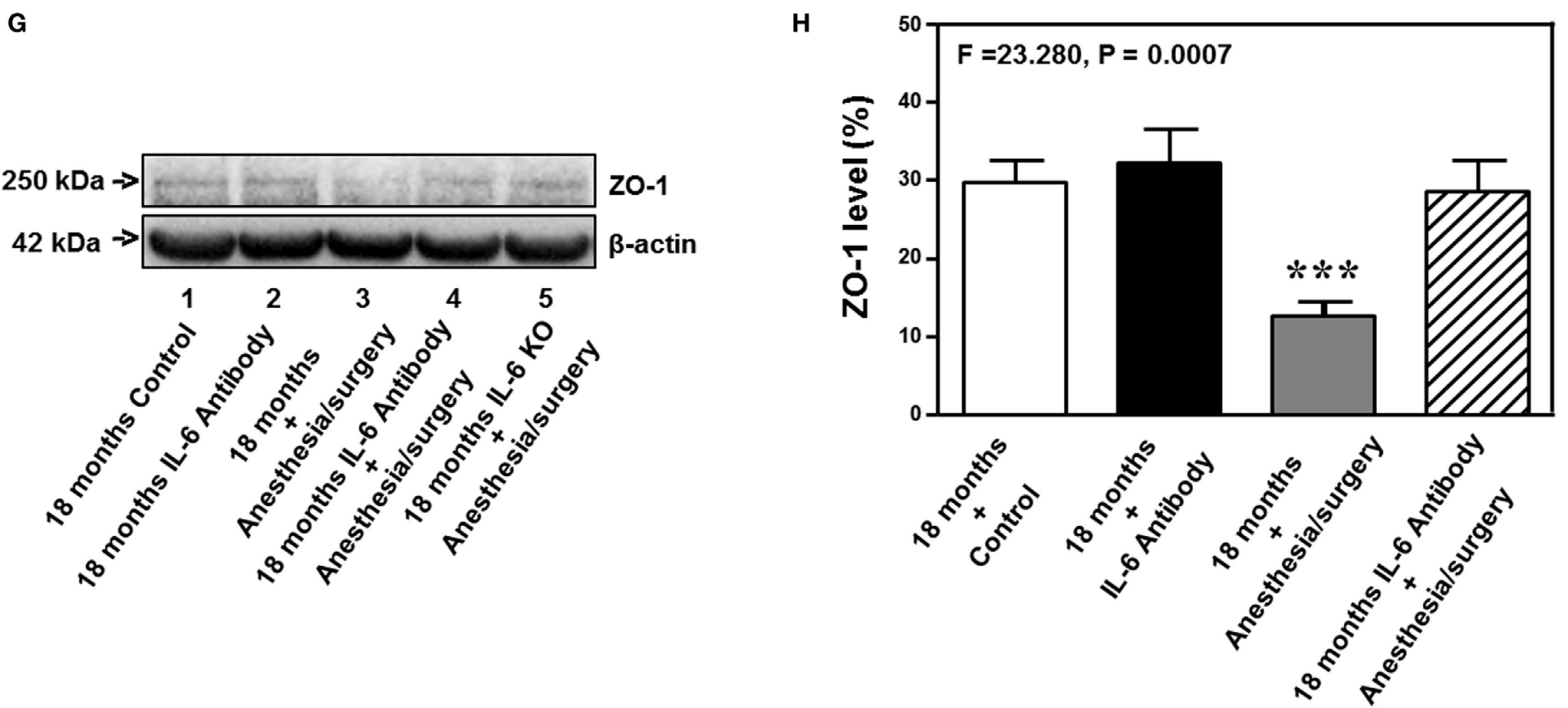
IL-6 antibody attenuates the anesthesia/surgery-induced reduction in cell junction proteins. The anesthesia/surgery reduces protein levels of β-catenin **(A,B)**, claudin **(C,D)**, occuludin **(E,F)**, and ZO-1 **(G,H)** in the hippocampus of 18-month-old mice as compared to control condition at 24 h after the anesthesia/surgery. Treatment of IL-6 attenuates these reductions in the levels of β-catenin **(A,B)**, claudin **(C,D)**, occuludin **(E,F)**, and ZO-1 **(G,H)** induced by the anesthesia/surgery. The knockout of IL-6 gene [lanes 5 in **(A,C,E,G)**] also attenuates the anesthesia/surgery-induced reduction in the levels of β-catenin **(A)**, claudin **(C)**, occludin **(E)**, and ZO-1 **(G)**. IL, interleukin. *N* = 6 in each group except IL-6 gene knockout group of **(A,C,E,G)**.

The 18-month-old mice received IL-6 antibody 18 h before the anesthesia/surgery and the hippocampus tissues were harvested 24 h after the anesthesia/surgery. Quantitative Western blot showed that the treatment of IL-6 antibody significantly attenuated the anesthesia/surgery-induced reduction in the levels of β-catenin (*F* = 7.420, *P* = 0.009, two-way ANOVA, Figures 8A,B), claudin (*F* = 10.310, *P* = 0.009, two-way ANOVA, Figures 8C,D), occludin (*F* = 10.340, *P* = 0.009, two-way ANOVA, Figures 8E,F), and ZO-1 (*F* = 23.280, *P* = 0.0007, two-way ANOVA, Figures 8G,H) in the hippocampus of the 18-month-old mice. The fifth band on each of the Western blot image represents the level of β-catenin (Figure 8A), claudin (Figure 8C), occludin (Figure 8E), and ZO-1 (Figure 8G) obtained from one 18-month-old IL-6 KO mouse. Taken together, these data suggest that treatment of IL-6 antibody or KO of IL-6 gene is able to attenuate the anesthesia/surgery-induced reduction in the levels of cell junction proteins.

## Discussion

Anesthesia and surgery (anesthesia/surgery) in rodents have been shown to induce cognitive impairment [(1–10, 33), reviewed in Ref. (11)]. However, the underlying mechanisms still remain largely to be determined. Given the fact that BBB dysfunction could contribute to cognitive impairment [(18, 46–48), reviewed in Ref. (17, 20)], we set out to investigate whether the anesthesia/surgery was able to induce BBB dysfunction, e.g., increase in BBB permeability and cognitive impairment in mice.

Dextran-tracer injection has been used to reveal BBB formation and function (15). We, therefore, employed dextran to assess the effects of the anesthesia/surgery on BBB function in our studies. The anesthesia/surgery induced BBB dysfunction as evidenced by the findings that the anesthesia/surgery increased the BBB permeability to 10-kDa dextran in the brain tissues of mice (Figures 2A,B). Moreover, there was greater BBB permeability to the 10-kDa dextran in the brain tissues of 18-month-old mice than that in the brain tissues of 9-month-old mice following the anesthesia/surgery: row 3 versus row 4 in Figure 2A; and gray bar versus net bar in Figure 2B. Finally, IL-6 antibody (Figures 2A,B) and knockout of IL-6 gene (Figure 2D) attenuated the anesthesia/surgery-induced increase in BBB permeability to 10-kDa dextran. The anesthesia/surgery may not induce the increase in BBB permeability to larger molecules, e.g., 70-kDa dextran. Finally, the anesthesia/surgery induced an age-associated increase in blood IL-6 levels (Figure 4). Collectively, these data support the hypothesis that the anesthesia/surgery induces an age-associated and IL-6 dependent increase in BBB permeability in mice.

Consistent with these data, we were able to show that the anesthesia/surgery induced cognitive impairment in the 18, but not 9, month-old mice (Figures 5 and 6). The data suggest that the anesthesia/surgery might cause an age-associated BBB dysfunction, leading to the age-associated cognitive impairment in rodents, pending on further investigation.

Neuroinflammation has been suggested to contribute to postoperative delirium [reviewed in Ref. (49)] and postoperative cognitive dysfunction [reviewed in Ref. (17)]. Specifically, IL-6 has been reported to be associated with learning and memory impairment in animals (50–52), cognitive dysfunction (53), mild cognitive impairment (54), and delirium in patients (55). In the current studies, we found that the anesthesia/surgery-induced an age-associated increase in blood IL-6 level (Figure 4); as well as an age-associated and IL-6-dependent BBB dysfunction (Figures 2 and 3). These findings further support the role of IL-6 in the neurotoxicity associated with anesthesia and surgery; and suggest that IL-6 may contribute to postoperative delirium and postoperative cognitive dysfunction *via* the impairment of BBB function. Finally, these results postulate the hypothesis that the accumulated IL-6, induced by the anesthesia and/or surgery, could attack the BBB, leading to BBB dysfunction.

The human IL-6 protein has 184 amino acids plus a 28-amino-acid-hydrophobic signal sequence (56). Blood IL-6 can be generated by circulating myeloid or lymphoid cells, or can be released from gut, liver, wound, muscle, and other local tissues [reviewed in Ref. (57)]. Specifically, IL-6 is produced in peripheral blood leukocytes, spleen, liver, kidney, and intestine (58, 59) and the induction of IL-6 generation is in nearly every human tissues and cells [reviewed in Ref. (57)]. A recent study showed that blood monocyte was able to generate IL-6 (60). The data from our current studies have established a system and shown that the anesthesia/surgery can increase blood IL-6 levels and may induce an IL-6-dependent increase in BBB permeability. However, the underlying mechanisms of such findings remain largely unknown. The future studies should assess whether injection of IL-6 is able to increase the BBB permeability in IL-6 KO mice. The future studies should also test a hypothesis that the anesthesia/surgery-induced increase in IL-6 may regulate junction protein levels, BBB permeability, and cognition in mice *via* cellular signal transduction, e.g., Wnt signaling pathway.

Anesthesia/surgery did not increase the BBB permeability of 70-kDa dextran (Figure 2E). The exact reason why the anesthesia/surgery increased the BBB permeability to 10-kDa dextran, but not 70-kDa dextran, is not known at the present time. The molecular weights of 70-kDa dextran are larger than that of 10-kDa dextran, it is conceivable that the anesthesia/surgery might only increase BBB permeability to small molecules (e.g., dextran, 10 kDa), but not large molecules (e.g., dextran, 70 kDa).

There are other studies that also demonstrate that anesthesia and surgery induce BBB dysfunction. Dittmar et al. showed that anesthetic isoflurane, administered after hypoxia, was able to induce apoptosis of endothelial cells *in vitro* (61). These findings suggest that isoflurane might damage BBB (61). Acharya et al. reported that anesthetic sevoflurane, but not isoflurane, was able to induce an aging-linked BBB compromise in rats, potentially leading to postoperative cognitive decline and later dementia (62). However, isoflurane was also reported to lead to hippocampal BBB compromise through changes in ultrastructure and occludin tight junction protein expression in aged rats (63). Finally, surgery (splenectomy) under anesthesia (2–3% isoflurane) was able to increase the BBB permeability measured with IgG immunohistochemistry in aged (22- to 23-month-old) rats (64). Consistent with these findings, the data from the current studies demonstrated that the surgery plus anesthesia (1.4% isoflurane up to 2 h) increased the BBB permeability to 10-kDa dextran. However, the data from the current studies further showed that minor surgery (opening and closing of abdominal cavity) under the anesthesia could induce an age-associated BBB dysfunction in mice (Figures 2 and 3). More importantly, the results from the current studies suggest that the anesthesia/surgery was able to selectively increase the BBB permeability to 10-kDa dextran, but not to 70-kDa dextran (Figure 2E). Finally, the findings from the current studies suggest that the anesthesia/surgery-induced increase in BBB permeability was dependent on IL-6 (Figures 2D and 3). The future studies will use the established system (the anesthesia/surgery-induced BBB permeability) to study whether BBB dysfunction contributes to postoperative cognitive dysfunction and postoperative delirium.

We found that the anesthesia/surgery was able to reduce the levels of tight junction protein claudin, occludin, and ZO-1 (Figures 7 and 8), but not adherent junction protein VE-cadherin, E-cadherin, and p120-catenin (Figure S1 in Supplementary Material). These data suggest that the anesthesia/surgery may selectively impair tight junction, but not adherent junction, leading to the increase in BBB permeability to small molecule, e.g., 10-kDa dextran, but not big molecule, e.g., 70-kDa dextran.

Interestingly, the anesthesia/surgery decreased level of β-catenin. β-catenin exists in three locations: cell membrane, cytosol, and nucleus (65, 66). The cell membrane β-catenin is one of the adherent junction proteins. However, the findings that the anesthesia/surgery increased the levels of phosphorylated β-catenin (Ser33/37/Thr41) suggest that the anesthesia/surgery may specifically decreased the levels of cytosol β-catenin, because the antibody (92 kDa, Cat: #9561, 1:1,000 dilution, Cell signaling, Danvers, MA, USA) we used in the current experiment specifically detected the phosphorylated β-catenin (Ser33/37/Thr41) levels in the cytosol (44). Nevertheless, it is still possible that the anesthesia/surgery could also regulate cell membrane and nucleus β-catenin. The future studies should specifically assess whether the anesthesia/surgery can also change the levels of cell membrane and nucleus β-catenin.

Taken together, the data obtained from the current studies suggest a hypothesized pathway that the anesthesia/surgery can increase plasma IL-6 levels, which regulate the metabolism of cytosol β-catenin, leading to reductions in the levels of tight junction proteins and increase in BBB permeability to small molecule. Consequently, the BBB dysfunction leads to cognitive impairment.

Recent studies have shown that CX3CR1+ monocytes can regulate learning and learning-dependent dendritic spine remodeling *via* TNF-α following infection with poly (I:C), a synthetic analog of double-stranded RNA (60). These data suggest that innate immune-cell activation may contribute to cognitive impairment. Thus, our future studies should include the investigation of the role of CX3CR1+ monocytes on the anesthesia/surgery-induced changes in BBB permeability, levels of junction protein, and cognition in mice.

There are several limitations of the current studies. First, we only assessed the effects of the anesthesia/surgery on BBB permeability in cortex of the mice. The effects of the anesthesia/surgery on BBB permeability in different brain regions (e.g., hippocampus) of the mice could be different. However, the outcomes from the current studies have established a system, which would be used to study the effects of anesthesia and/or surgery on BBB permeability in other brain regions. Second, our present study cannot define cellular sources and targets of the plasma IL-6. What cells release IL-6 and how this affects cells in the neurovascular unit warrant further investigation. Third, we did not use littermate controls for IL-6 KO mice throughout the experiments. We will use the littermate controls for IL-6 KO mice in the future investigation. Finally, we only assessed the effects of the anesthesia/surgery on BBB permeability in cortex to establish a system in the current studies. In the future investigations, we will use the established system to determine the effects of the anesthesia/surgery on BBB permeability in other brain regions (e.g., hippocampus) and the potential association with the observed behavioral changes.

In conclusion, the current studies mainly established a system in adult and older mice to study the effects of abdominal surgery under isoflurane anesthesia (anesthesia/surgery) on BBB permeability and behavioral changes. The data only suggest that the anesthesia/surgery may increase the BBB permeability to 10-kDa dextran, but not 70-kDa dextran, in the cortex of mice. This anesthesia/surgery-induced increase in the BBB permeability to dextran could be dependent on IL-6 and might be greater in older mice (e.g., 18-month-old mice). Moreover, the anesthesia/surgery induced an age-associated cognitive impairment in the mice. Given the fact that BBB dysfunction is associated with cognitive impairment, the results from the current studies suggest that the anesthesia/surgery would induce postoperative delirium and cognitive impairment by damaging BBB function, pending further investigation. These findings would promote further studies of the BBB-associated underlying mechanisms of postoperative delirium and postoperative cognitive dysfunction.

## Ethics Statement

All experiments were performed in accordance with the National Institutes of Health guidelines and regulations. The animal protocol was approved by the Massachusetts General Hospital (Boston, MA, USA) Standing Committee on the Use of Animals in Research and Teaching.

## Author Contributions

ZX, SY, CG, GY, YS, YZ, XF, and EL conceived and designed the project. SY, CG, EM, EE, YD, and YZ performed all the experiments and prepared the figures. ZX, SY, and CG wrote the manuscript. All authors reviewed the manuscript.

## Conflict of Interest Statement

The authors declare that the research was conducted in the absence of any commercial or financial relationships that could be construed as a potential conflict of interest.
