# Supplementary material for: Anesthesia and Surgery Impair Blood–Brain Barrier and Cognitive Function in Mice
Source: Front Immunol. 2017 Aug 9;8:902. doi: 10.3389/fimmu.2017.00902 (PMC5552714; doi:10.3389/fimmu.2017.00902)
Supplement: Supplementary file 1 [file Data_Sheet_1.DOCX]

**Anesthesia and surgery impairs blood brain barrier and cognitive function in mice**

Siming Yang, Changping Gu, Emiri T. Mandeville, Yuanlin Dong, Elga Esposito, Yiying Zhang, Guang Yang, Yuan Shen, Xiaobing Fu, Eng H. Lo and Zhongcong Xie

**Supplement Figure**

**
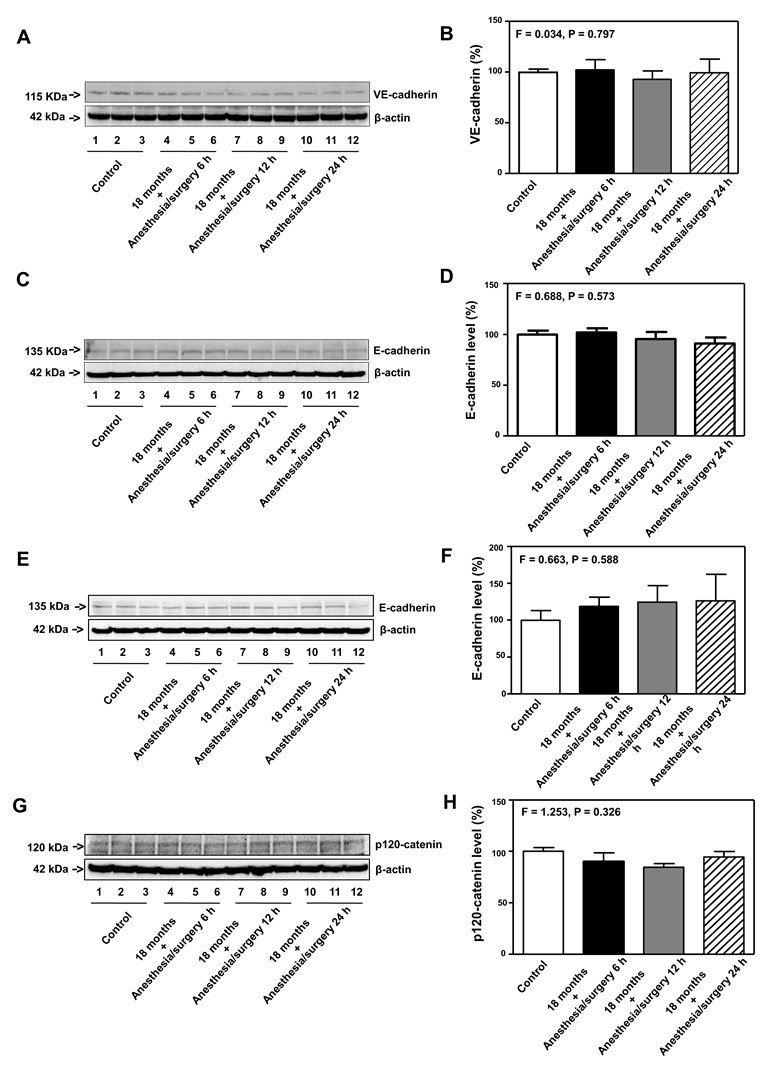
**

**Supplement Figure Anesthesia/surgery has no effect on the levels of adherent junction proteins.**Anesthesia/surgery has no effect on the levels of VE-cadherin in hippocampus (Figure **A** and **B),** E-cadherin in cortex (Figure **C** and **D),** E-cadherin in hippocampus (Figure **E** and **F),** p120-catenin in hippocampus (Figure **G** and **H)** of 18-month-old mice at 6, 12, 24 hours after the anesthesia/surgery as compared to the control condition. N = 6 in control or anesthesia/surgery group.
